# AFG2A-related encephalopathy, expanding the neurodevelopmental and epileptic spectrum

**DOI:** 10.1186/s13023-026-04204-w

**Published:** 2026-04-03

**Authors:** Laia Nou-Fontanet, Gaetano Cantalupo, Frederik Braun, Alia Ramírez Camacho, Verónica González Álvarez, Verónica Delgadillo Chilavert, Víctor Soto Insuga, Luisa Arrabal Fernández, Itziar Alonso-Colmenero, Alexis Arzimanoglou, Carmen Fons

**Affiliations:** 1 Early-Onset and Genetic Epilepsies Unit, Neurology and Neurophysiology Department, Hospital Sant Joan de Déu, University of Barcelona, Esplugues de Llobregat, Barcelona, Spain; 2 Complex Epilepsies Research Group, Institut de Recerca de Sant Joan de Déu, Esplugues de Llobregat, Barcelona, Spain; 3 Innovation Biomedicine Section, Department of Engineering for Innovation Medicine, University of Verona, Verona, Italy; 4 Child Neuropsychiatry Unit, University Hospital of Verona, Verona, Italy; 5 Center for Research on Epilepsy in Pediatric Age (CREP), University Hospital of Verona, Verona, Italy; 6 Institute of Human Genetics, University Hostpital Essen, University Duisburg-Essen, Essen, Germany; 7 Neurology Department, Hospital Niño Jesús, Madrid, Spain; 8 Neurology Department, Hospital Universitario Virgen de las Nieves, Granada, Spain; 9 Centro de Investigación Biomédica en Red de Enfermedades Raras (CIBERER), Instituto de Salud Carlos III (ISCIII), Madrid, Spain

**Keywords:** SPATA5, AFG2A developmental and epileptic encephalopathy, Infantile epileptic spasms syndrome, Microcephaly, Deafness

## Abstract

**Objectives:**

To expand the clinical features, epilepsy phenotype, and genotype in individuals with AFG2A-related encephalopathy (AFG2A-RE), previously known SPATA5-related encephalopathy, and to explore potential associations between genotype and epilepsy manifestations.

**Methods:**

We conducted a systematic literature review focusing on AFG2A-RE publications (45 patients), also included 6 of our patients recently reported as the result of a multicentre, retrospective, observational, and descriptive study. Available clinical descriptions, electroencephalography and MRI data, metabolic screening, genetic findings, and treatment responses were assessed.

**Results:**

A total of 51 individuals with AFG2A-RE were included, 29 males; mean age 8.35 years. The most frequently described clinical features included intellectual disability (97.92%), hearing loss (93.62%), microcephaly (85.71%), visual impairment (79.49%), hypotonia (71.74%), spasticity (60.87%), and movement disorders (36.96%). Epilepsy was present in 74.71% of cases, with seizures of generalized onset being the most common (70.83%), and infantile epileptic spasms syndrome (IESS) was the predominant epilepsy syndrome at onset (66.67%). Epilepsy was often drug-resistant (82.35%). Brain MRI abnormalities were frequently observed (68.29%), including hypomyelination (39.02%), brain atrophy (34.15%), and a thin corpus callosum (29.27%).

**Significance:**

This review presents the largest cohort of individuals with AFG2A-RE reported to date. AFG2A-RE is an ultra-rare, recessive disorder, sometimes presenting as a developmental and epileptic encephalopathy (DEE), characterised by the triad of epilepsy, congenital microcephaly, and deafness, and typically associated with intellectual disability, spasticity, and movement disorders. The cardinal clinical features of AFG2A-RE may mimic those of a mitochondrial disease. No significant associations between genotype and epilepsy phenotype were observed.

**Supplementary Information:**

The online version contains supplementary material available at 10.1186/s13023-026-04204-w.

## Background

AFG2A-related encephalopathy (AFG2A-RE), previously known as SPATA5-related encephalopathy, is a rare neurodevelopmental disorder characterized by intellectual disability, microcephaly, hearing loss, seizures, and brain abnormalities (OMIM #616577). First reported by Tanaka et al. in 2015, this disorder is caused by rare biallelic pathogenic variants in the *AFG2A* gene. The gene encodes an AAA ATPase involved in mitochondrial dynamics, ribosome assembly, and cellular homeostasis, and its dysfunction has been linked to proteotoxic stress and genomic instability. Approximately 73% of affected individuals develop epilepsy, which is often drug-resistant, yet detailed information on seizure types and treatment responses remain limited. This study aims to characterize the neurodevelopmental spectrum, describe the epilepsy phenotype, and investigate potential genotype-phenotype associations in a large cohort of individuals with AFG2A-RE.

## Introduction

AFG2A-related encephalopathy (AFG2A-RE), previously known as SPATA5-related encephalopathy, is a neurodevelopmental disorder typically associated with hearing loss, seizures, and brain abnormalities (NEDHSB; OMIM #616577). It was first described by Tanaka et al. in 2015 [1], who reported 14 individuals from 10 families presenting with microcephaly, intellectual disability, hypotonia, spasticity, seizures, sensorineural hearing loss, and cortical visual impairment, all of whom carried rare biallelic pathogenic variants in the *AFG2A* gene. Since then, up to 43 affected individuals have been reported in the literature as single cases or small series [2–10].

The *AFG2A* gene (OMIM *613940), located in the chromosomal region 4q28.1, encodes the AAA ATPase homolog A, and was previously known as *SPATA5* (spermatogenesis-associated protein 5) or *SPAF* (spermatogenesis-associated factor). Although initially linked to spermatogenesis, *AFG2A* is now recognized as having a broader biological role. It is expressed in multiple tissues including the testes, spleen, skin, intestines, brain, and skeletal muscle [8]. Although its function is not fully elucidated, current evidence supports its involvement in mitochondrial function [4, 10, 10, 11], ribosome assembly and protein synthesis [12, 13]. Recent findings indicate that AFG2A is part of the protein complex 55LCC, composed of AAA+ ATPases AFG2A (SPATA5)- AFG2B (formerly known as SPATA5L1), together with heterodimeric partners C1orf109-CINP [13, 14]. This complex plays a key role in the cytoplasmic maturation of the 60S ribosomal subunit and contributes to the maintenance of DNA replication integrity and genome stability, particularly under stress conditions. Dysfunction of the 55LCC complex has been associated with proteotoxic stress and chromosomal instability, suggesting a crucial role in maintaining cellular homeostasis [14].

Approximately 73% of the individuals with AFG2A-RE present with epilepsy, most of whom had drug-resistant epilepsy (DRE) [6]. However, neither the epilepsy phenotype nor the response to anti-seizure medications (ASMs) have been widely described.

The aims of our study are to provide a comprehensive description of the clinical and neurodevelopmental spectrum and the epilepsy phenotype, in a large series of individuals with AFG2A-RE, and to investigate potential associations between genotype and epilepsy phenotype.

## Subjects, materials and methods

We conducted a systematic literature review of available data from individuals with AFG2A-RE, following the PRISMA methodology [15]. The last search was performed in July 2025. The literature search was conducted in MEDLINE via Ovid and PubMed, BioMed Central, Cochrane, Free Medical Journals, and Medscape, and was limited to digitally available articles without restrictions on publication language. The free-text search terms included: [(SPATA5) OR (NEDHSB) OR (EHLMRS) OR (OMIM #616577) OR (SPATA5 related encephalopathy) OR (AFG2A) OR (AFG2A related encephalopathy]. Inclusion criteria for previously published individuals were: (1) confirmed diagnosis of AFG2A-RE, and (2) availability of genetic variants and clinical information in the publication. A PRISMA flow chart outlining the study selection process is provided as Supplementary Fig. 1.

To the 45 previously published cases, we added data from 6 new cases via a multicentric, descriptive, observational, and retrospective study. New individuals were recruited from European Reference Network for rare and complex epilepsies (ERN-EpiCARE) centres and the Spanish Society of Pediatric Neurology through calls for participation. We included new individuals with characteristic clinical features of AFG2A-RE, who harbored two homozygous or compound heterozygous variants in the AFG2A gene. Genetic diagnosis was performed via clinical whole-exome sequencing, and variants were confirmed by Sanger sequencing. Data collected included age, sex, perinatal period, anthropometric measurements, neurologic and extra-neurologic symptoms, and, regarding the epilepsy phenotype, age at seizure onset, seizure types, anti-seizure medications (ASMs) and their effects, and EEG abnormalities. Brain MRI findings, metabolic analysis, and gene variants in *AFG2A* were also collected. Variant pathogenicity was classified according to the American College of Medical Genetics and Genomics/Association of Molecular Pathology (ACGM) guidelines [16]. The *AFG2A* transcript NM_145207.3 was used for coding variant nomenclature.

Statistical analysis were performed using the SPSS v0.24.0 statistical package (IBM Corp., Armonk, NY, USA). Descriptive statistics were used to summarize the characteristics of the study population. Categorical variables were presented as frequencies and percentages, while continuous variables were reported as mean ± standard deviations. Associations between categorical variables were assessed using Fisher’s Exact Test due to the small sample size. A two-tailed p-value < 0.05 was considered statistically significant.

## Results

**Fig. 1:**
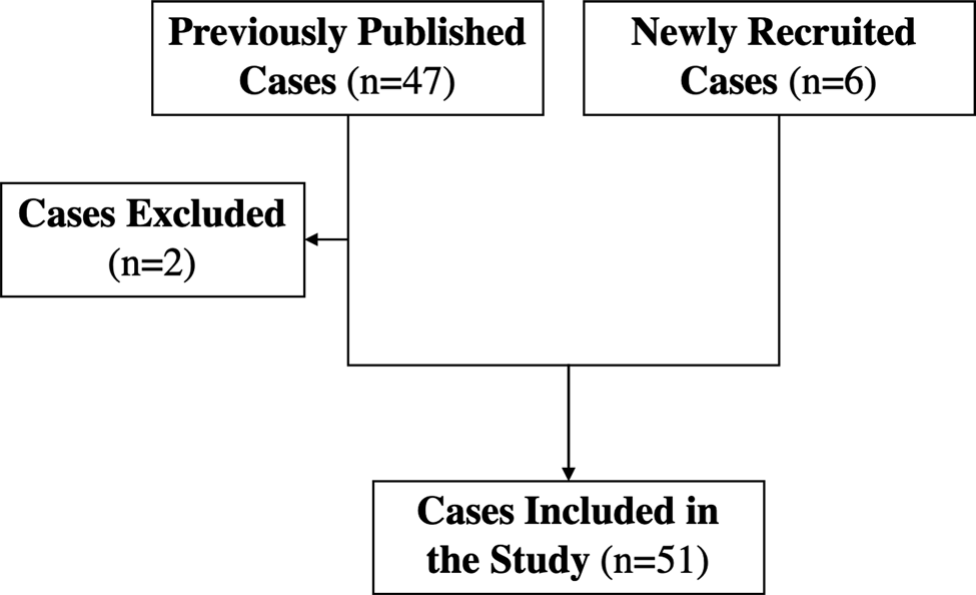
Diagram representing the individuals included in the study

This review includes 45 previously reported individuals and 6 newly recruited individuals with AFG2A-RE. Twelve of the 13 articles published between 2015 and 2025 were included in the review. Two previously published individuals were excluded: one due to missing genetic information and one because the case was reported twice (see Fig. 1). A detailed description of the reviewed publications is provided in Supplementary Table 1.

## Population characteristics

Twenty-nine individuals were males (56.7%). The mean age at inclusion was 8.35 years (range: 9 months − 41 years). All the individuals reported were alive, except for three (4.89%) who died at the ages of 3, 4.9 and 13.4 years (Patient ID 26, 27 and 45). The cause of death of the third was respiratory failure. The causes of death for the other two individuals were not described in the respective publications.

## Clinical phenotype spectrum

The detailed clinical phenotype, epilepsy characteristics, and genetic variants are summarised in Table 1.

**Table 1:** Summary of the genetic variants, pathogenicity prediction, and main clinical symptoms

Patient ID	Patient ID Publication	Sex (M/F) Age (y)	AFG2Avariants DNA change; protein change	Type of Genetic variation (in silico pathogenicity prediction)	Microcephaly	Neurodevelopmental Delay	Intellectual Disability	Epilepsy	Deafness	Visual Impairment	Gastrointestinal Impairment	Cardiologic Impairment	Motor Impairment
1	Tanaka, 2015–1	F 6	c0.2531C > T; p.Ala844Val c0.1574_1578del; p.Asn525Thrfs*20	Missense (P) Frameshift (P)	+	+	+	-	+	+	+	NA	+
2	Tanaka, 2015–2	F 9	c0.2531C > T; p.Ala844Val c0.1574_1578del; p.Asn525Thrfs*20	Missense (P) Frameshift (P)	+	+	+	+	+	+	+	NA	+
3	Tanaka, 2015–3	F 5	c0.2362_2371delATTGATAGAA;p.Ile788Serfs*47 c0.251 G > A; p.Arg84Gln	Frameshift (P) Missense (P)	+	+	+	+	+	+	+	NA	+
4	Tanaka, 2015–4	F 4	c0.1343C > T; p.Ser448Leu c0.556C > T; p.Arg186*	Missense (LP) Nonsense (P)	+	+	+	+	+	+	+	NA	+
5	Tanaka, 2015–5	F 2	c0.298 G > A; p.Ala100Thr c0.556C > T; p.Arg186*	Missense (P) Nonsense (P)	+	+	+	+	+	+	+	NA	+
6	Tanaka, 2015–6	M 11	c0.2351 G > A; p.Arg784Gln c0.269 G > T; p.Ser90Ile	Missense (P) Missense (LP)	+	+	+	+	+	+	+	NA	+
7	Tanaka, 2015–7	F 6	c0.2351 G > A; p.Arg784Gln c0.269 G > T; p.Ser90Ile	Missense (P) Missense (LP)	+	+	+	+	+	NA	+	NA	+
8	Tanaka, 2015–8	M 19	c0.1883A > G; p.Asp628Gly c0.1586 G > A; p.Arg529Gln	Missense (P) Missense (P)	NA	+	+	+	+	+	+	NA	+
9	Tanaka, 2015–9	F 14	c0.1883A > G; p.Asp628Gly c0.1586 G > A; p.Arg529Gln	Missense (P) Missense (P)	+	+	+	+	+	+	-	NA	+
10	Tanaka, 2015–10	M 8	c0.1878 G > C; p.Trp626Cys c0.1714+1 G > A; p.?	Missense (P) Splice donor+2 (P)	+	+	+	+	+	+	+	NA	+
11	Tanaka, 2015–11	M 3	c0.1878 G > C; p.Trp626Cys c0.1714+1 G > A; p.?	Missense (P) Splice donor+2 (P)	+	+	+	+	+	+	+	NA	+
12	Tanaka, 2015–12	M 5	c0.1883A > G; p.Asp628Gly c0.1677C > A; p.Tyr559*	Missense (P) Nonsense (LP)	-	+	+	+	+	+	+	NA	+
13	Tanaka, 2015–13	F 11	c0.989_991del; p.Thr330del c0.989_991del; p.Thr330del	Frameshift (P) Frameshift (P)	+	+	+	+	+	+	+	NA	+
14	Tanaka, 2015–14	M 4	c0.251 G > A; p.Arg84Gln c0.251 G > A; p.Arg84Gln	Missense (P) Missense (P)	+	+	+	+	+	+	+	NA	+
15	Kurata, 2016–1	M 4	c0.989_991del; p.Thr330del c0.2130_2133del; p.Glu711Profs*21	Frameshift (P) Deletion (P)	+	+	NA	+	+	+	-	-	+
16	Kurata, 2016–2	F 2	c0.989_991del; p.Thr330del c0.2130_2133del; p.Glu711Profs*21	Frameshift (P) Deletion (P)	+	+	NA	+	+	NA	-	-	+
17	Kurata, 2016–3	M 5	c0.967T > A; p.Phe323Ile c0.2146 G > C; p.Ala716Pro	Missense (VUS) Missense (LP)	+	+	NA	+	+	NA	+ GER	+ HF	+
18	Buchert, 2016 -V1	M 22	c0.2081 G > A; p.Gly694Glu c0.2081 G > A; p.Gly694Glu	Missense (P) Missense (P)	+	-	+	-	NA	NA	NA	NA	NA
19	Buchert, 2016-V2	M 20	c0.2081 G > A; p.Gly694Glu c0.2081 G > A; p.Gly694Glu	Missense (P) Missense (P)	+	-	+	-	+	NA	NA	NA	NA
20	Buchert, 2016-V3	M 12	c0.2081 G > A; p.Gly694Glu c0.2081 G > A; p.Gly694Glu	Missense (P) Missense (P)	+	-	+	-	NA	NA	NA	NA	+
21	Buchert, 2016-V4	M 5	c0.2081 G > A; p.Gly694Glu c0.2081 G > A; p.Gly694Glu	Missense (P) Missense (P)	+	-	+	-	NA	NA	NA	NA	NA
22	Buchert, 2016-V5	M 2	c0.2081 G > A; p.Gly694Glu c0.2081 G > A; p.Gly694Glu	Missense (P) Missense (P)	+	-	+	-	NA	NA	NA	NA	NA
23	Buchert, 2016-IV12	M 41	c0.2081 G > A; p.Gly694Glu c0.2081 G > A; p.Gly694Glu	Missense (P) Missense (P)	+	-	+	-	+	+	+ CC	NA	+
24	Buchert, 2016-IV13	M 39	c0.2081 G > A; p.Gly694Glu c0.2081 G > A; p.Gly694Glu	Missense (P) Missense (P)	+	-	+	-	+	+	+ CC	NA	+
25	Buchert, 2016-B1	M 1.4	c0.1822_1824de; p.Asp608del c0.1822_1824del; p.Asp608del	Frameshift (LP) Frameshift (LP)	+	+	+	**+**	+	+	NA	+ ASD	+
26	Puusepp, 2018-a-1	F 4 (d)	c0.250C > T; pArg84* c0.989_991del; p.Thr330del	Nonsense (LP) Frameshift (P)	+	+	+	+	+	NA	NA	NA	+
27	Puusepp, 2018-a-2	M 3 (d)	c0.250C > T; pArg84* c0.989_991del; p.Thr330del	Nonsense (LP) Frameshift (P)	+	+	+	+	+	NA	+ MHSM	NA	+
28	Puusepp, 2018-a-3	F 9	c0.554 G > A; p.Gly185Glu c0.989_991del; p.Thr330del	Missense (P) Frameshift (P)	+	+	+	+	+	+	+ RG	NA	+
29	Puusepp, 2018-a-4	M 3	c0.394C > T; p.Gln132* c0.989_991del; p.Thr330del	Nonsense (P) Frameshift (P)	+	+	+	+	+	+	+ GER	NA	+
30	Puusepp, 2018-a-5	F 5	c0.700C > T; p.Gln234* c0.2384C > G; p.Pro795Arg	Nonsense (LP) Missense (LP)	+	+	+	+	+	+	+ GER &CC	NA	+
31	Szczaluba, 2017–1	F 4	c0.1714+1 G > A; p.Thr339del c0.1714+1 G > A; p.Thr339del	Splice site (P) Splice site (P)	+	+	+	+	+	+	+ CC	-	+
32	Szczaluba, 2017–2	F 1	c0.1714+1 G > A; p.Thr339del c0.1714+1 G > A; p.Thr339del	Splice site (P) Splice site (P)	-	-	NA	-	+	-	-	NA	+
33	Papuc, 2019–47651	M 13	c0.2081_2082del; p.Gly694Phefs*23 c0.989_991del; p.Thr330del	Donor (P) Non frameshift (P)	-	**+**	+	**+**	+	+	NA	NA	+
34	Papuc, 2019–73068	M 6	c0.2389C > G; p.Pro797Ala c0.1A > C, p.Met1Leu	Missense (LP) Start Loss (P)	+	+	+	**+**	+	+	NA	NA	+
35	Zanus, 2020	M 8	c0.1942A > G; p.Lys648Glu c0.1942A > G; p.Lys648Glu	Missense (VUS) Missense (VUS)	+	+	+	**+**	+	+	NA	NA	+
36	Khurana, 2019	NA NA	c0.1069 G > T; p.Gly357* c0.304C > T; p.Pro102Ser	Nonsense (P) Missense (LP)	NA	+	+	+	+	NA	NA	NA	NA
37	Braun, 2021	F 10	c0.394C > T; p.Gln132* c0.989_991del; p.Thr330del	Nonsense (P) Non frameshift (P)	+	+	+	-	+	-	-	-	+
38	Stancheva, 2020–1	M 12	c0.554 G > A; p.Gly185Glu c0.1831C > T; p.Pro611Ser	Missense (P) Missense (LP)	-	**+**	+	-	-	-	NA	NA	+
39	Stancheva, 2020–2	F 1	c0.554 G > A; p.Gly185Glu c0.1831C > T; p.Pro611Ser	Missense (P) Missense (LP)	+	+	+	-	-	-	NA	NA	+
40	Raggio, 2023	M 10	c0.2004del; p.Pro669fs c0.2293 G > A; p.Asp765Asn	Nonsense (P) Missense (VUS)	+	+	+	+	+	+	NA	-	+
41	Nou, 2025 -1	M 7	c0.251 G > A; p.Arg84Gln c0.251 G > A; p.Arg84Gln	Missense (P) Missense (P)	+	+	+	+	+	+	+ GER	-	+
42	Nou, 2025 -2	F 4	c0.251 G > A; p.Arg84Gln c0.1586 G > A; p.Arg529Gln	Missense (P) Missense (P)	+	+	+	+	+	+	+ GER &DP	-	+
43	Nou, 2025 -3	M 7	c0.251 G > A; p.Arg84Gln c0.1064T > C; p.Ile355Thr	Missense (P) Missense (P)	+	+	+	+	+	+	+ DP	-	+
44	Nou, 2025 -4	M 8	c0.251 G > A; p.Arg84Gln c0.2174T > C; p.Phe725Ser	Missense (P) Missense (LP)	+	+	+	**+**	+	-	+ DP	-	+
45	Nou, 2025 -5	M 13 (d)	c0.327_328del; p.Lys110fs c0.1631T > G; p.Leu544Arg	Frameshift (LP) Missense (LP)	+	+	+	**+**	+	+	+ GER &DP	-	+
46	Present study-1	F 2	c0.1833_1839de; p.Ser612Ter c0.1159C > T; p.Pro387Ser	Frameshift (LP) Missense (VUS)	-	**+**	+	**+**	+	-	+ GER &DP	-	+
47	Present study-2	F 2	c0.989_991del; p.Thr330del c0.2166_2176del; p.Ile723	Donor (LP) Donor (LP)	+	+	+	+	+	NA	+ CC	+	+
48	Present study-3	F 3	c0.251 G > A; p.Arg84Gln c0.1523C > T; p.Ala508Val	Missense (P) Missense (LP)	-	**+**	+	-	+	-	NA	-	+
49	Present study-4	F 3	c0.989_991del; p.Thr330del c0.1714+1 G > A; p.Thr339del	Donor (LP) Splice site (P)	+	+	+	+	+	+	+ CC	-	+
50	Present study-5	M 18	c0.1942A > G; p.Lys648Glu c0.554 G > A; p.Lys648Glu	Missense (VUS) Missense (LP)	-	+	+	+	-	-	-	-	+
51	Present study-6	M 1	c0.251 G > A; p.Arg84Gln c0.2130_2133del; p.Glu711Profs*21	Missense (P) Donor (LP)	+	+	+	+	+	+	+ DP	-	+

Abbreviations: Male (M) Female (F), Years (y), Dead (d), Gastrointestinal impairment (GII), Cardiologic impairment (CI), Motor impairment (MI), Pathogenic (P), Likely pathogenic (LP), Variant of uncertain significance (VUS), Yes (+), No (-), Not available (NA), Gastroesophageal reflux (GER), Heart Failure (HF), Chronic constipation (CC), Atrial septal defect (ASD), Death (d), Mild hepatosplenomegaly (MHSM), Recurrent gastroenteritis (RG), Delayed gross motor development (DGMD) and Dysphagia (DP)

Perinatal data were available for 32 cases, with 75% reporting no complications. The most frequent complication was neonatal hypotonia (12.5%), followed by neonatal respiratory distress syndrome (9.38%), moderate-to-late preterm birth (6.25%), and intrauterine growth restriction (6.25%).

The vast majority of the individuals presented with microcephaly (85.71%). Growth data was available for 30 individuals, with most showing normal growth (73.33%). However, short stature (13.33%) and failure to thrive (13.33%) were also reported.

Neurodevelopmental delay was a predominant feature (84.31%). Almost all individuals had intellectual disability (ID) (97.92%). Data on ID severity were available for 32 cases, revealing a wide spectrum: moderate-to-severe in 25%, and severe-to-profound in 75%. Absence of speech was reported in all individuals with ID. The only individual without ID was a toddler with delayed gross motor development (Patient ID 32). Motor impairment was present in almost all individuals with available data (97.83%). The most common motor symptoms were hypotonia (71.74%) and spasticity (60.87%). Non-paroxysmal movement disorders were present in 36.96% of the individuals, with dystonia being the most prevalent (21.74%), followed by dyskinesia (6.52%), ataxia (6.52%), chorea (4.35%), and athetosis (2.17%). All individuals were assessed using the Gross Motor Function Classification System (GMFCS): 59.09% were classified as level V, 27.27% as level IV, 9.09% as level III, and 4.55%. as level II.

Almost all individuals in the study exhibited hearing loss (93.62%). Visual impairment was also highly prevalent (79.49%), among those reported cortical blindness (9.68%), abnormality of refraction (9.68%), and strabismus (9.68%) were reported.

**Fig. 2:**
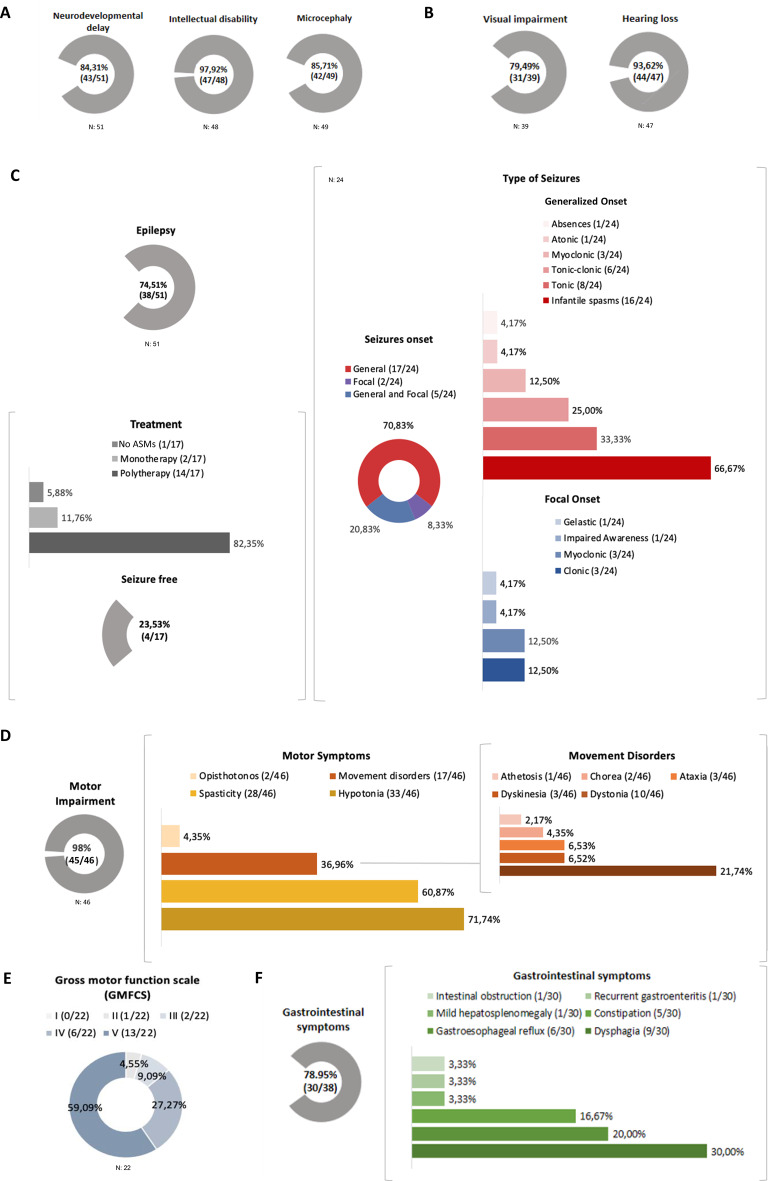
Clinical spectrum of symptoms of individuals with AFG2A-RE and frequencies. N indicates the number of patients with information available. A. Neurodevelopmental outcomes: neurodevelopmental delay, intellectual disability, and microcephaly. B. Sensory impairments: visual impairment, and hearing loss. C. Epilepsy: seizure types, ASM, and seizure control. D. Motor impairment: type of motor symptoms, and movement disorders. E. Gross motor function: GMFCS classification. F. Gastrointestinal manifestations: type of gastrointestinal symptom. Abbreviations: Antiseizure medications (ASMs).

A high proportion of individuals (78.95%) experienced gastrointestinal symptoms. Dysphagia was the most common symptom (30%), followed by gastroesophageal reflux (20%), and constipation (16.67%), and mild hepatosplenomegaly (3.33%), recurrent gastroenteritis (3.33%), and intestinal obstruction (3.33%). The clinical phenotypic spectrum is depicted in Fig. 2.

## Epilepsy phenotype spectrum

Thirty-eight out of 51 individuals presented with epilepsy. Detailed data were available for 24 individuals. Among these 24 individuals, generalized seizures were the most common (70.84%), while focal seizures were observed in two individuals; five individuals presented both types of seizures. Infantile epileptic spasms syndrome (IESS) was reported in 16 individuals (66.67%). The mean age at IESS onset of was 9.53 months (range: 2.5–27). Eight individuals (33.44%) experienced tonic seizures, 6 tonic-clonic (25%), 3 myoclonic (12.5%), 1 atonic (4.17%), and 1 atypical absence (4.17%). Focal seizures were documented in 3 individuals, characterized by clonic (12.5%) or myoclonic (12.5%) semiology, while focal impaired consciousness seizures and gelastic seizures were both observed in only one individual. Three individuals (12.5%) were reported to have seizures with complex ocular movements, with one case lacking EEG correlation.

EEG abnormalities were recorded in 44 individuals, with detailed descriptions available for 20. Interictal epileptiform activity was present in 95% of individuals.

Multifocal epileptiform discharges were observed in 75%, seven of whom (35%) had a focal predominance. Hypsarrhythmia was recorded in four individuals (20%), and photoparoxysmal activity in one individual (5%).

Data on ASM treatment were available for 17 individuals, most of whom were receiving polytherapy (82.35%). Detailed epilepsy follow-up was available for only 16 individuals, with only four reported as seizure free. Three of these individuals, who presented with IESS and generalized tonic-clonic seizures, received polytherapy: lacosamide and levetiracetam (Patient ID 25); valproate, levetiracetam and ACTH (Patient ID 31); lamotrigine and valproate (Patient ID 50). The fourth individual (Patient ID 41), with refractory IESS, became seizure free with the ketogenic diet (KD) and is now on KD monotherapy. KD treatment was also administered to four other individuals (Patient ID 35, 42–44), showing variable seizure reduction rates: 0% in two cases (Patient ID 35, 44), 30% in one (Patient ID 43), and 70% in another (Patient ID 42). Greater seizure control was observed in individuals who initiated KD early. Improvements in social interaction were noted in one individual (Patient ID 42), while attentional and motor functions improved in two (Patient ID 43–44). The epilepsy phenotypic spectrum is summarized in Table 2.

**Table 2:** Summary of the individuals with epilepsy. In this table, type of seizure at epilepsy onset, electroencephalogram information, anti-seizure therapies and outcomes

Patient ID	Age (months), seizure type at onset	Other seizure types	EEG description	ASMs (Seizure control)
2	NA, NA	NA	Abnormal EEG. NA.	NA (NA)
3	NA, NA	NA	Abnormal EEG. NA.	NA (NA)
4	NA, NA	NA	Abnormal EEG. NA.	NA (NA)
5	NA, NA	NA	Abnormal EEG. NA.	NA (NA)
6	NA, NA	NA	Abnormal EEG. NA.	NA (NA)
7	NA, NA	NA	Abnormal EEG. NA.	NA (NA)
8	NA, NA	NA	Abnormal EEG. NA.	NA (NA)
9	NA, NA	NA	Abnormal EEG. NA.	NA (NA)
10	NA, NA	NA	Abnormal EEG. NA.	NA (NA)
11	NA, NA	NA	Abnormal EEG. NA.	NA (NA)
12	NA, NA	NA	Abnormal EEG. NA.	NA (NA)
13	NA, NA	NA	Abnormal EEG. NA.	NA (NA)
14	NA, NA	NA	Abnormal EEG. NA.	NA (NA)
15	14, IESS	GMTS	Abnormal EEG. Interictal Epileptiform Activity: Brief bursts of diffuse spike-wave complexes with low synchronicity, multifocal spikes or spikes/polyspikes with occipital dominance).	PB, VPA (refractory epilepsy)
16	14, IESS	NA	Abnormal EEG. Interictal Epileptiform Activity: sharp or slow waves which evolved into diffuse periodic spike bursts with occipital and frontal dominance	NA (NA)
17	6, IESS	GMTS	Abnormal EEG. Interictal Epileptiform Activity: multifocal spikes with occipital dominance.	PB, ZNS, VPA (refractory epilepsy)
25	8, IESS	NA	Abnormal EEG. Interictal Epileptiform Activ: Hypsarrhythmia	LCM, LEV (seizure free)
26	NA, NA	GTCS	Abnormal EEG. Interictal Epileptiform Activity: multifocal epileptiform discharges	NA (NA)
27	NA, NA	FMMS	Abnormal EEG. Interictal Epileptiform Activity: multifocal epileptiform discharges (sharp-wave)	NA (NA)
28	NA, NA	FMCS, GTCS	Abnormal EEG. Interictal Epileptiform Activity: multifocal epileptiform discharges (spikes)	NA (NA)
29	NA, NA	GMTS, FMMS	Abnormal EEG. Interictal Epileptiform Activity: Hypsarrhythmia.	NA (NA)
30	NA, NA	FMMS	Abnormal EEG. Interictal Epileptiform Activity: multifocal epileptiform discharges (polyspikes).	NA (NA)
31	14, IESS	Convergent strabismus with eyeballs tremor	Abnormal EEG. Interictal Epileptiform Activity: multifocal epileptiform discharges (burst of sharp and slow wave complexes, spikes, and polyspike-wave complexes).	VPA, LEV, ACTH (seizure free)
33	8, IESS	GMTS, Absences, FMCS, GMAS, gelastic seizures.	Abnormal EEG. NA.	NA (NA)
34	5, IESS	GMTS, GTCS, FICS.	Abnormal EEG. NA.	NA (NA)
35	6, IESS	Subtle seizures with nystagmus, staring, and hyporeactivity persisted in daily clusters.	Abnormal EEG. Generalized slow activity and disorganized. Interictal Epileptiform Activity: slow multifocal spikes and waves of high-amplitude discharge with occipital dominance) Ictal: interruption of background slow and paroxysmal activity and by emergence of a prolonged condition characterized by the appearance of short sequences of low-voltage beta activity, moving from one hemisphere to the other in concomitance with subtle seizures with ocular movements.	ACTH, VGB, TPM, ZNS, LTG, PB, KD (refractory epilepsy)
36	NA, NA	NA	NA	NA (NA)
40	3, IESS	GMTS, GTCS, GMMS	Abnormal EEG. Generalized slow activity and disorganized. IEA: Hypsarrhythmia.	LTG, CBD (refractory epilepsy)
41	27, IESS	N	Abnormal EEG. Interictal Epileptiform Activity: multifocal epileptiform discharges	VPA, VGB, ACTH, ESM, CLB, KD (refractory epilepsy)
42	7, IESS	N	Abnormal EEG. Generalized slow activity and disorganized. Interictal Epileptiform Activity: Hypsarrhythmia.	ACTH, VGB, VPA, LEV, ZNS, CZP, LCM, PER, KD (seizure free)
43	14, IESS	N	Abnormal EEG. NA.	VGB,ACTH, CLB, PER, TPM, VPA, LTG, KD (seizure free)
44	6, GMTS	IS	Abnormal EEG. Interictal Epileptiform Activity: multifocal epileptiform discharges	LEV, VPA, LTG, RFM, KD (seizure free)
45	14, IESS	N	Abnormal EEG. IEA: MED.	VPA, VGB, CLB, PB, LTG (seizure free)
46	NA, NA	GMMS	Abnormal EEG. Generalized slow activity and disorganized. Interictal epileptiform abnormalities: monomorphic slow waves ± spike-waves 2.5 Hz and 300 mV with bilateral fronto-centro-temporal dominancy.	LEV (seizure free)
47	14, NA	GTCS	Abnormal EEG. NA.	CLB (seizure free)
49	2.5, IESS	GMMS, FMCS (hemiclonic), GMTS.	Abnormal EEG. Generalized slow activity and disorganized. Interictal epileptiform abnormalities: multifocal spikes with right centro-temporal dominance, generalized spike and waves. Photo-paroxysmal.	BRV, NTZ, ACZ, (previous LEV, VPA, LTG, CLB, TPM) (seizure free)
50	7.2, GTCS	N	Abnormal EEG.	LTG, VPA (refractory epilepsy)
51	7, IESS	N	Abnormal EEG. Interictal epileptiform abnormalities: spikes, polyspikes, sharp waves, spike-wave complex and polyspike-wave.	VGB, ACTH (seizure free)

Abbreviations: Electroencephalogram (EEG), Anti-seizure medications (ASMs), Ketogenic diet (KD), Not available (NA). Infantile Epileptic Spasms Syndrome (IESS), General Motor Tonic Seizure (GMTS), General Tonic-Clonic Seizure (GTCS), Focal Motor Myoclonic Seizure (FMMS), Focal Motor Clonic Seizure (FMCS), General Motor Atonic Seizure (GMAS), Focal Impairment Consciousness Seizure (FICS), General Motor Myoclonic Seizure (GMMS). Phenobarbital (PB), Valproate (VPA), Zonisamide (ZNS), Lacosamide (LCM), Levetiracetam (LEV), Adrenocorticotropic hormone (ACTH), Vigabatrin (VGB), Topiramate (TPM), Lamotrigine (LTG), Cannabidiol (CBD), Ethosuximide (ESM), Clobazam, (CLB), Rufinamide (RFM), Perampanel (PER), Carbamazepine (CZP), Brivacetam (BRV), Acetazolamide (ACZ), Nitrazetam (NTZ)

## Metabolic analysis

Two individuals in our cohort (Patient ID 43, 45) exhibited a persistent mild elevation of lactate (1.9–3.9 mmol/l) without other abnormalities in mitochondrial biomarkers, including acid-base balance, creatine kinase, pyruvate, organic acids, amino acids, carnitines, oxidative stress markers, circulating cytokines, or mitochondrial growth factors. No elevated lactate was detected in urine or cerebrospinal fluid.

## Neuroimaging

The most frequently reported MRI abnormalities were hypomyelination (39.02%) (Patient IDs 3, 5, 8, 15–17, 26–27, 29, 33–34, 38, 40, 45–46), global brain atrophy (34.15%) (Patient IDs 6, 8, 12, 15, 17, 24, 26–27, 33, 37, 42, 44, 46–47), and a thin corpus callosum (29.27%) (Patient IDs 1–2, 15–17, 29, 33, 35, 37–38, 40, 46). Less commonly described findings included leukoencephalopathy (7.32%) (Patient IDs 30, 37, 42, 45, 49), cerebral white matter atrophy (7.32%) (Patient IDs 30, 34–35, 45, 49), and small basal ganglia (4.88%) (Patient IDs 26, 46). Brain MRI was normal in 31.71% of cases.

### *AFG2 A*gene variants

**Fig. 3:**
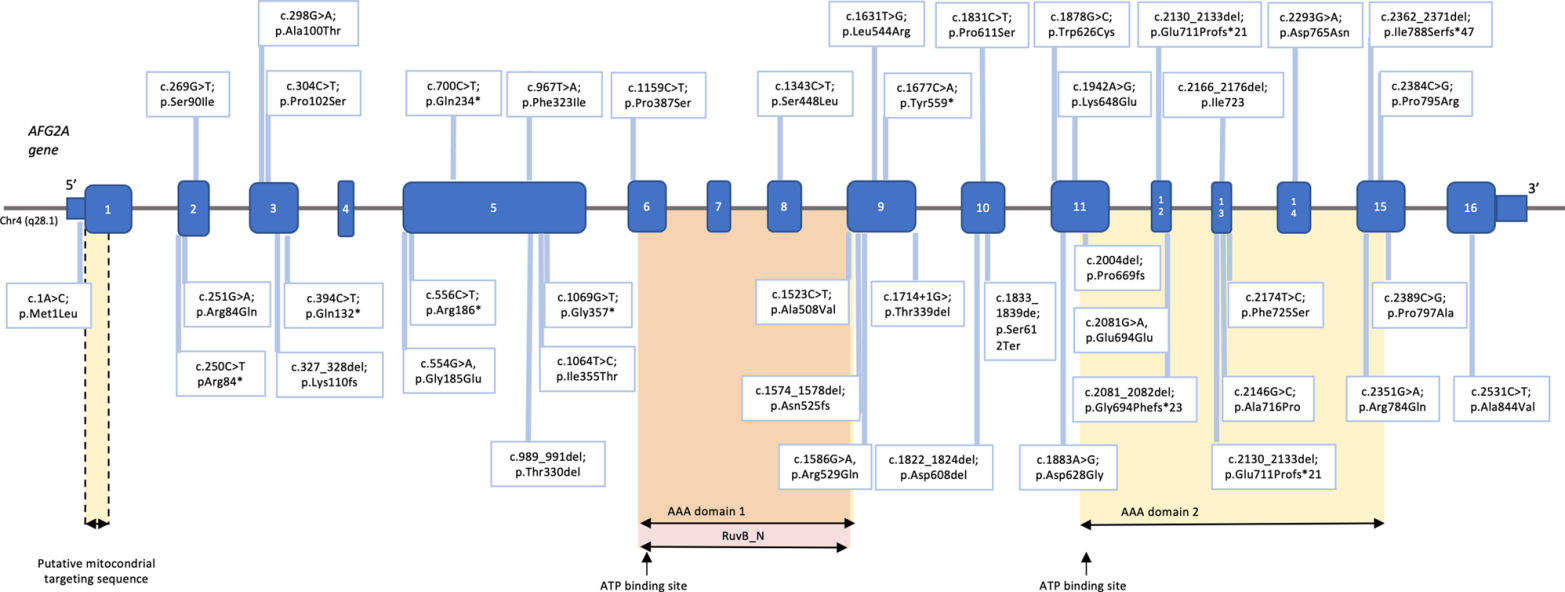
Representation of *AFG2A* gene variants with protein coding domains

Forty-two different *AFG2A* gene variants had been reported in the 51 included individuals (Fig. 3). The majority exhibited compound heterozygous variants (86.28%). All *AFG2A* gene variants were located in exons except one intronic variant, reported in single individual. The most frequent *AFG2A* gene variants were c0.2081 G > A (13.73%), c0.989_991del (11.76%), c0.251 G > A (9.80%) and c0.1714+1 G > A (6.86%). Most of the variants were missense (58.82%), with others being frameshift (14.71%), nonsense (9.8%), deletion (6.68%), splice site/splice donor (6.68%), non-frameshift (1.96%) and start loss (0.98%).

Regarding pathogenicity, variants were classified into pathogenic (72.55%), likely pathogenic (21.57%) and variant of uncertain significance (5.88%). The exon distribution of variants was as follows: 1–5’UTR (0.98%), 2 (13.73%), 3 (4.9%), 5 (21.57%), 6 (0.98%), 8 (0.98%), 9 (14.7%), 10 (4.9%), 11 (8.82%), 12 (14.71%), 13 (5.88%), 14 (0.98%), 15 (4.9%), and 16 (1.96%). Some variants were located within the AAA domain 1 and RuvB_N domain (1.96%), and AAA domain 2 (26.47%).

## Association between epilepsy and genetic variants

The c0.2081 G > A variant was associated with absence of epilepsy (*p* < 0.001). However, all individuals carrying this missense variant belonged to a single family and were homozygous for it (Patient IDs 19–24). No significant association was observed between epilepsy and the three other most frequent recurrent variants (c0.989_991del, c0.251 G > A and c0.1714+1 G > A), nor between epilepsy and variant type (missense, frameshift, deletion, splice site, or nonsense). Therefore, these results should be interpreted with caution.

## Discussion

This review represents the most comprehensive clinical and genetic characterization to date of AFG2A-RE, encompassing 51 individuals, including 6 newly reported cases and a detailed review of the 45 previously published cases. Our findings further expand the clinical spectrum of this neurodevelopmental disorder, with particular emphasis on the epilepsy phenotype.

Consistent with previous reports, the most prevalent clinical features included ID, sensorineural hearing loss, microcephaly, and visual impairment [1]. Motor impairment was nearly universal, with hypotonia and spasticity as the most common findings. Interestingly, over one-third of individuals exhibited movement disorders, with dystonia being the most frequent. As seen in other developmental and epileptic encephalopathies (DEE), movement disorders are likely to be under-recognized and under-reported [17]. These findings support the classification of AFG2A-RE as a complex neurodevelopmental disorder with multisystem involvement.

Epilepsy is a major hallmark of AGF2A-RE, affecting up to 75% of individuals. As a novel finding that expands the epilepsy phenotype, we report that IESS is the predominant form of epilepsy presentation, expanding the epilepsy phenotype previously attributed to AGF2A-RE. In patients with early-onset epilepsy, especially those presenting with IEES, and associated developmental impairment, the phenotype supports classification as a DEE [18]. As in other DEEs, epilepsy is frequently drug-resistant, with most individuals requiring polytherapy and only a minority achieving seizure control. As we recently reported, individuals with IESS do not achieve seizure control with usual first-line treatment (neither corticosteroids nor vigabatrin). The KD showed partial efficacy in selected cases, particularly when initiated early. One individual achieved seizure freedom with KD monotherapy, and three others experienced improvements not only in seizure burden but also in behavioral and motor domains. These findings suggest that KD may be a therapeutic option in AFG2A-RE, especially given the limited efficacy of conventional ASMs [11]. EEG findings remain insufficiently characterized, as available descriptions are limited to a very small number of individuals, making it difficult to establish any specific or consistent EEG pattern (Patient ID 15–17, 25–31, 35, 40–46, 49–51).

Neuroimaging findings were abnormal in approximately two-thirds of individuals, most commonly showing white matter abnormalities, global brain atrophy, and thin corpus callosum.

Over 40 variants in *AFG2A* have been described as causative of AFG2A-RE. The most frequent variants are c0.2081 G > A, c0.989_991del, c0.251 G > A, and c0.1714+1 G > A, present in 42% of the individuals, and more than half of the variants are missense. Due to the low prevalence of AFG2A-RE, establishing associations between different genetic variants and epilepsy profiles remains challenging. Although the c0.2081 G > A variant was associated with absence of epilepsy, all carriers belonged to a single consanguineous family, limiting the generalizability of this association. Further studies in larger populations are needed to explore potential relationships between genotype and phenotype, considering both disease severity and epilepsy phenotype.

The cardinal clinical features of AFG2A-RE mimic those of mitochondrial diseases. In particular, deafness is a common trait in mitochondrial disorders, as is visual impairment due to optic atrophy. Indeed, both the auditory and optic nerves are highly vulnerable to energy defects. Although AFG2A deficiency leads to mitochondrial dysfunction, as our group and others have previously published [4, 10, 11], no plasma and urine mitochondrial biomarker has yet been identified. To date, no consistent biochemical alterations have been reported, except for occasional mild increases in plasma lactate (as observed in Nou, 2025 – cases 3 and 5), without other mitochondrial-related abnormalities.

The other pathogenic role of the AFG2A protein as part of the 55LCC complex is further supported by the fact that individuals with AFG2B-related encephalopathy present a clinical phenotype similar to those with AFG2A-RE. Pathogenic variants in *AFG2B,* previously named *SPATA5L1,* are associated with a neurodevelopmental disorders characterized by overlapping clinical features, including ID, neurodevelopmental delay, epilepsy, hearing loss, and dystonia [19]. These phenotypic similarities between AFG2A-RE and AFG2B-RE reinforce the hypothesis that the 55LCC complex plays a critical role in neurodevelopment, underlying a shared molecular basis for both conditions. In contrast, no neurological disorders have been linked to the other components of the complex, C1orf109 and CINP, to date.

The prevalence of AFG2A-RE has been estimated at around 3% of early-onset developmental and epileptic encephalopathies [6]. This disorder is likely underdiagnosed, despite exhibiting clinical features that constitute a hallmark of AFG2A-RE (microcephaly, deafness, and DEE).

This study has limitations inherent to the retrospective design, including incomplete data, variable follow-up duration, and potential publication bias in the literature review. Additionally, given that AFG2A-RE is a rare disease, the statistical power to detect genotype-phenotype correlations was limited. Prospective natural history studies will be essential to gain a deeper understanding of AFG2A-RE.

## Conclusion

In conclusion, AFG2A-RE is a recessive genetic disease characterized by a distinctive triad of IESS, microcephaly, and hearing loss, typically accompanied by ID and motor impairment. No significant genotype-phenotype correlations regarding epilepsy were identified. Prospective natural history studies across multiple reference centres are needed to advance understanding of AFG2A-RE, refine genotype-phenotype correlations, and guide the development of targeted therapies.

## Electronic supplementary material

Below is the link to the electronic supplementary material.

Supplementary Material 1

Supplementary Material 2

## Data Availability

All data generated or analysed during this study are included in this published article and its supplementary information files. Additional anonymised data that support the findings of this study are available from the corresponding author on reasonable request.

## Abbreviations

AFG2A-RE: AFG2A-related encephalopathy
AFG2B: AFG2 AAA ATPase HOMOLOG B
ASM/ASMs: Anti-Seizure Medication(s)
DEE: Developmental and Epileptic Encephalopathy
DRE: Drug-Resistant Epilepsy
EEG: Electroencephalogram
ERN-EpiCARE: European Reference Network for rare and complex epilepsies
GMFCS: Gross Motor Function Classification System
ID: Intellectual Disability
IESS: Infantile Epileptic Spasms Syndrome
KD: Ketogenic Diet
MRI: Magnetic Resonance Imaging
NEDHSB: Neurodevelopmental Disorder with Hearing Loss, Seizures, and Brain Abnormalities
OMIM: Online Mendelian Inheritance in Man
SPAF: Spermatogenesis-Associated Factor
SPATA5: Spermatogenesis-Associated Protein 5
UTR: Untranslated Region
C1orf109: Chromosome 1 open reading frame 109, also known as AFG2 Interacting Ribosome Maturation Factor
