# Supplementary material for: AFG2A-related encephalopathy, expanding the neurodevelopmental and epileptic spectrum
Source: Orphanet J Rare Dis. 2026 Apr 3;21:128. doi: 10.1186/s13023-026-04204-w (PMC13049760; doi:10.1186/s13023-026-04204-w)
Supplement: Supplementary file 1 — Supplementary Material 1 [file 13023_2026_4204_MOESM1_ESM.docx]

**Supplementary Table 1.** Description of the articles included in the systematic review

| **Author, year** | **Country** | **Study type** | **Patients included**  **(total number)** * |
| --- | --- | --- | --- |
| Tanaka, 2015 | USA | Case series | 14 (14) |
| Kurata, 2016 | Japan | Case series | 3 (3) |
| Buchert, 2016 | Germany | Case series | 8 (8) |
| Puusepp, 2018 | Estonia | Case series | 5 (5) |
| Szczaluba, 2017 | Poland | Case series | 2 (2) |
| Papuc, 2019-a | Switzerland | Case series | 0 (1)^1^ |
| Papuc, 2019-b | Switzerland | Case series | 2 (3)^2^ |
| Zanus, 2020 | Italy | Case report | 1 (1) |
| Khurana, 2019 | USA | Case report | 1 (1) |
| Braun, 2021 | German | Case report | 1 (1) |
| Stancheva, 2020 | Bulgaria | Case series | 2 (2) |
| Raggio, 2023 | Chile | Case report | 1 (1) |
| Nou, 2025 | Spain | Case series | 5 (5) |

* The patients included in this review are indicated as patients included, and the total number of patients in each publication is shown in brackets. ^1^ Patient excluded because it is also published in Papuc, 2019-b. ^2^ Patient excluded because genetic information was not complete.
