# Supplementary material for: AFG2A-related encephalopathy, expanding the neurodevelopmental and epileptic spectrum
Source: Orphanet J Rare Dis. 2026 Apr 3;21:128. doi: 10.1186/s13023-026-04204-w (PMC13049760; doi:10.1186/s13023-026-04204-w)
Supplement: Supplementary file 2 — Supplementary Material 2 [file 13023_2026_4204_MOESM2_ESM.docx]

**Identification of studies via databases and registries**

Records removed *before screening*:

Duplicate records removed
(n = 1)

Records marked as ineligible by automation tools (n = 0)

Records removed for other reasons (n = 1)

Records identified from*:

Databases (n = 37)

Registries (n = 0)

**Identification**

Records screened

(n =35)

Records excluded**

(n =22)

Reports sought for retrieval

(n =13)

Reports not retrieved

(n =0)

**Screening**

Reports excluded: (n=0)

Reports assessed for eligibility

(n = 13)

Studies included in review

(n =13)

Reports of included studies

(n =13)

**Included**

Supplementary Figure 1. PRISMA flow diagram illustrating the process of identification, screening, eligibility assessment, and inclusion of studies in the review.
